# Data from four consecutive cohorts of students in Australia (2019–2022) show the impact of the COVID-19 pandemic on domestic and international university students’ mental health

**DOI:** 10.1177/00048674241233111

**Published:** 2024-02-21

**Authors:** Genevieve A Dingle, Rong Han, Sakinah SJ Alhadad, Emma Beckman, Sarah V Bentley, Sjaan R Gomersall, Leanne Hides, Fiona Maccallum, Blake M McKimmie, Kalina Rossa, Simon S Smith, Zoe C Walter, Elyse Williams, Olivia Wright

**Affiliations:** 1 School of Psychology, The University of Queensland, St Lucia, QLD, Australia; 2 School of Education and Professional Studies, Griffith University, Nathan, QLD, Australia; 3 School of Human Movement and Nutrition Sciences, The University of Queensland, St Lucia, QLD, Australia; 4 School of Health and Rehabilitation Sciences, The University of Queensland, St Lucia, QLD, Australia; 5 Lives Lived Well Research Group, The University of Queensland, St Lucia, QLD, Australia; 6 Institute for Social Science Research, The University of Queensland, St Lucia, QLD, Australia; 7 ARC Centre of Excellence for Children and Families Over the Life Course, Indooroopilly, QLD, Australia

**Keywords:** COVID-19, university students, international students, mental health, wellbeing, depression, anxiety, somatic

## Abstract

**Introduction:**

COVID-19 and related travel and social restrictions caused significant stress for university students in Australia and globally. Learning quickly moved online and many students (particularly international students) were separated from social and economic support. This study examined the impact of the pandemic from pre-pandemic (2019) to the COVID-19 Omicron wave (2022) on domestic and international students’ mental health.

**Methods:**

Participants were 1540 students (72% females, 28% international) in four first-year cohorts (2019, 2020, 2021, 2022). We screened for mental health concerns (% positive) and symptom scores for depression, anxiety and somatic distress using the PsyCheck, and general wellbeing using the Warwick–Edinburgh Mental Well-being scale.

**Results:**

From pre-COVID (2019) to the first wave of COVID-19 (2020), the proportion of students screening positive for mental health problems rose in both domestic students (66–76%) and international students (46–67%). Depression symptoms and wellbeing were worse in 2020 than in 2019, 2021 and 2022. Anxiety symptoms increased from 2019 to 2020 and continued to rise in 2021 and 2022. Somatic symptoms did not show an effect of cohort. Contrary to expectations, domestic students reported higher distress and lower wellbeing than international students across cohorts.

**Conclusion:**

The pandemic was associated with a marked increase in psychological distress in first-year university students, not all of which settled with the easing of restrictions. Post-pandemic recovery in the Australian university sector must include university-wide access to mental health information and support for incoming students.

The first year of university is an exciting time of learning and personal growth, when students typically become more independent from their families and form new relationships with friends and colleagues. Despite the positive aspects of this transition, however, many students find it difficult to adapt to a new educational and social environment. National data are not routinely collected on experiences of mental ill-health among university students and there is a lack of Australian-based research (Orygen, 2017). However, a 2010 survey of 6479 Australian university students found that nearly one in five experienced mental health problems, and two thirds suffered from symptoms of distress (Stallman, 2010). The National Tertiary Student Wellbeing Survey 2016 reported similar findings, with 65% of young adult students reporting high or very high psychological distress (Rickwood et al., 2016). When tertiary students were compared with non-students in three national surveys (Cvetkovski et al., 2012), there was higher prevalence of moderate distress in tertiary students than non-students in the Household, Income and Labour Dynamics in Australia (wave 7) survey and the 2007 National Survey of Mental Health and Wellbeing, but not in the 2007–2008 National Health Survey. Cvetkovski and colleagues’ study did not make a distinction between tertiary students enrolled as domestic or international students, although it might be expected that these two groups of students experience the transition to university in different ways.

International students are those who are identified during the enrolment process as not being born in Australia and/or holding an Australian citizenship. International students comprise an increasingly larger proportion of students in Australia, growing from around 120,000 to around 950,000 in the two decades from 1999 to 2019 (Department of Education, 2020). Around half of these are enrolled in universities and the rest in school, vocational training or English language intensive courses for overseas students (ELICOS) institutions. A comparison of international and domestic students’ mental health and wellbeing is warranted because, while they share the usual transitional challenges with domestic students, international students are additionally relocating and adjusting to a new climate, social and educational cultures (Van Gijn-Grosvenor and Huisman, 2020) and, for some, a new language (Rosenthal et al., 2006). Previous research on international students’ wellbeing in Australia has tended to recruit samples comprising only of international students (e.g. Forbes-Mewett and Sawyer, 2016; Rosenthal et al., 2006), making it impossible to compare between international and domestic students. Furthermore, the few comparative studies have reported inconsistent findings. For example, one study found that while international students in Australia had significantly lower social support, used more dysfunctional coping strategies and had greater mismatched expectations compared to domestic students, psychological distress did not differ between international and domestic students (Khawaja and Dempsey, 2008). In contrast, another study found that international students, particularly male students, were at increased risk of several adverse health outcomes while also being less likely to seek help for mental health and related problems (Skromanis et al., 2018).

Further complicating our understanding of the topic, in 2020, students’ transitions to university were disrupted when the coronavirus disease (COVID-19) became a global pandemic. In Australia, the National Cabinet attempted to halt the spread of COVID-19 by introducing international travel restrictions and mandating quarantine periods for people allowed to enter the country. In 2020, it was estimated that more than 100,000 Chinese students (the most common country of origin for international students in Australian universities) planning to study in Australia were unable to travel (Ziguras and Tran, 2020). Many international students who remained in Australia experienced financial hardship with the rapid loss of casual jobs from the hospitality and retail sectors, and no access to federal Government support, such as the Job Seeker allowance. For those students who were onshore (domestic and international), public health measures including social distancing restrictions required an immediate shift to online learning. Virtual classes (many of which were pre-recorded and viewed in students’ own time) offered fewer opportunities for the friendly and informal discussions that may occur when students interact in and between classes. Students reported an increase in loneliness and a decrease in sense of belonging at university (Dingle et al., 2022). Most university educators were not adequately trained in effective online teaching methods and were also working from home without the level of technological support required (e.g. Keane et al., 2022), so the quality of teaching and learning was less certain than previously. Unsurprisingly, some students experienced a loss of motivation for learning and an increase in psychological distress (Cao et al., 2020; Fruehwirth et al., 2021).

This is the only Australian study to document the mental health of first-year university students over four consecutive cohorts during the pandemic, and to compare domestic and international students. The 2019 cohort students were surveyed before COVID-19, the 2020 cohort students were surveyed during the first wave of COVID-19 when strict lockdowns were in place and the 2021 and 2022 cohorts were surveyed during the Delta and Omicron strains of coronavirus when travel restrictions and social distancing measures were gradually eased in Australia. Specific hypotheses were:

*H1.* The COVID-19 pandemic will be associated with a detrimental effect on students’ mental health, such that the proportion of participants screening positive for mental health problems and the average number of depression, anxiety and somatic symptoms will be higher and wellbeing scores will be lower in the 2020 and subsequent cohorts (2021, 2022) than in the 2019 cohort.
*H2.* Due to the additional causes of stress for international students such as travel restrictions, casual job losses and lack of access to Australian Government support, there will be an effect of enrolment status such that international students will report significantly more psychological distress and lower wellbeing than their domestic student counterparts during the COVID-19 pandemic.

## Methods

### Participants

Characteristics of the 1540 university students (73.9% female, 27.8% international, mean age = 20.1 years) are presented by year cohort and enrolment status group in Table 1. Using the nine broad ethnicity categories used by the Australian Bureau of Statistics, the most common ethnic groups represented were North-East Asian (24.4%), South-East Asian (13.5%) and Southern and Central Asian (6.1%). There were some age differences between domestic and international students across the cohorts. In 2019, international students (21.6 years) were older than domestic students (18.9 years), but this trend reversed over subsequent cohorts. Most participants were single (67.3%), followed by being in a relationship but not living together (23.4%). Across cohorts, most domestic students were living with family, while the living situation of international students was more varied. In 2019, most international students were living away from home in a share house or some form of student accommodation. During 2020 and 2021, more international students were living with family (i.e. studying from offshore), and in 2022 most international students were again living in shared student accommodation.

**Table 1.** Demographic characteristics of the 1540 first-year university students, analysed by cohort and enrolment status.

	2019 cohort		2020 cohort		2021 cohort		2022 cohort	
	Domestic *N* = 293 % in brackets	International *N* = 179 % in brackets	Domestic *N* = 301 % in brackets	International *N* = 98 % in brackets	Domestic *N* = 266 % in brackets	International *N* = 99 % in brackets	Domestic *N* = 251 % in brackets	International *N* = 50 % in brackets
Gender								
Female	216 (73.7)	112 (62.6)	223 (74.1)	72 (73.4)	201 (75.6)	77 (77.8)	198 (78.9)	38 (76.0)
Male	76 (25.9)	63 (35.1)	76 (25.2)	25 (25.5)	63 (23.7)	21 (21.2)	46 (18.3)	11 (22.0)
Non-binary	N/A	N/A	N/A	N/A	2 (0.8)	1 (1.0)	7 (2.8)	0
Prefer not to say	1 (0.3)	4 (2.2)	2 (0.7)	1 (1.0)	0	0	0	1 (2.0)
Age (M, SD)	18.9 (3.4)	21.6 (3.2)	19.0 (3.7)	21.1 (5.5)	20.8 (6.7)	20.2 (1.9)	20.8 (5.8)	19.7 (2.1)
Relationship status								
Single	193 (65.9)	124 (69.3)	216 (71.8)	64 (65.3)	165 (62.0)	73 (73.7)	159 (63.3)	40 (80.0)
In relationship (not living together)	71 (24.2)	39 (21.8)	67 (22.3)	26 (26.5)	63 (23.7)	23 (23.2)	63 (25.1)	8 (16.0)
Living together	25 (8.5)	13 (7.3)	9 (3.0)	6 (6.1)	19 (7.1)	3 (3.0)	20 (8.0)	1 (2.0)
Married	4 (1.4)	4 (2.2)	8 (2.7)	0	15 (5.6)	0	6 (2.4)	0
Divorced	0	0	1 (0.3)	1 (1.0)	2 (0.8)	0	2 (0.8)	0
Other	0	0	0	1 (1.0)	2 (0.8)	0	1 (0.4)	1 (2.0)
Living situation								
Living with family	184 (62.8)	6 (3.4)	239 (79.4)	25 (25.5)	162 (60.9)	81 (81.8)	154 (61.4)	8 (16.0)
Living alone	13 (4.4)	11 (6.1)	14 (4.7)	10 (10.2)	15 (5.6)	7 (7.1)	19 (7.6)	3 (6.0)
Share house	44 (15.0)	65 (36.3)	25 (8.3)	19 (19.4)	42 (15.8)	3 (3.0)	36 (14.3)	7 (14.0)
UQ college	36 (12.3)	13 (7.3)	14 (4.7)	5 (5.1)	29 (10.9)	1 (1.0)	29 (11.6)	9 (18.0)
Student accommodation (shared)	10 (3.4)	47 (26.3)	5 (1.7)	26 (26.5)	11 (4.1)	4 (4.0)	10 (4.0)	7 (14.0)
Student accommodation (studio)	5 (1.7)	38 (21.2)	4 (1.3)	13 (13.3)	7 (2.6)	3 (3.0)	3 (1.2)	16 (32.0)

N/A: not applicable; SD: standard deviation; M: mean; UQ: University of Queensland.

Participants were recruited via several purposive sampling methods. Members of the research team gave brief talks about student mental health and the study to large first-year lectures in several Schools of the University and at the university-based English language institute. We also placed advertisements on the first-year students’ social media sites at the University, on the School of Psychology research participation scheme and via word of mouth at two residential colleges on campus that are known to have large enrolments of international students. The researchers also contacted international student associations and invited them to advertise the survey.

### Measures

The online survey was developed in consultation with an international student administrator and a group of international student advisors to ensure that the wording was easily understood by students who spoke English as an additional language.

#### Mental health screening

The PsyCheck mental health screener (Jenner et al., 2013) is a 20-item measure that is quick to administer and assesses symptoms of depression and anxiety and somatic symptoms such as headaches or upset stomach. This was considered important because research indicates that many international students report somatic symptoms rather than emotional or cognitive expressions of depression and anxiety (Chang et al., 2017). Participants responded *Yes* = 1 or *No* = 0 to 20 questions asking whether they have experienced each symptom in the last 30 days. Endorsed items are summed to provide a score out of 20. Scores above 5 indicate a positive screen, indicating a recommendation for further assessment and management of a mental health issue. The internal consistency of the PsyCheck measure in our study was good, with a Cronbach’s α = 0.88.

*Depression* was indexed by four items on the PsyCheck: *Do you feel unhappy? Do you find it difficult to enjoy your daily activities? Are you unable to play a useful part in life? Do you feel that you are a worthless person?* These were the highest loading items on the ‘depression’ factor in an exploratory factor analysis on the PsyCheck using the 2019 data, that also represent key diagnostic criteria for depression (anhedonia and sad mood). Respondents were asked whether they had experienced each symptom over the past 30 days, scoring *yes* = 1 or *no* = 0. Item scores were summed to produce a score in the range of 0–4. The depression scale showed adequate reliability in our sample (Cronbach’s α = 0.75).

*Anxiety* was indexed by a score computed from the sum of scores on four PsyCheck items: *Are you easily frightened? Do your hands shake? Do you feel nervous? Do you find it difficult to make decisions?* These were the highest loading items on the ‘anxiety’ factor in an exploratory factor analysis on the PsyCheck using the 2019 data. Respondents were asked whether they had experienced each symptom over the past 30 days, scoring *yes* = 1 or *no* = 0. Item scores were summed to produce a score in the range of 0–4. The anxiety subscale showed adequate reliability in our sample (Cronbach’s α = 0.60).

*Somatic* distress was indexed by a score computed from the sum of scores on four PsyCheck items: *Do you often have headaches? Is your appetite poor? Is your digestion poor? Do you have uncomfortable feelings in the stomach?* These were the highest loading items on the ‘somatic’ factor in an exploratory factor analysis on the PsyCheck using the 2019 data. Respondents were asked whether they had experienced each symptom over the past 30 days, scoring *yes* = 1 or *no* = 0. Item scores were summed to produce a score in the range of 0–4. The somatic subscale showed adequate reliability in our sample (Cronbach’s α = 0.61).

#### Wellbeing

The Short Warwick–Edinburgh Mental Well-being Scale (Ng Fat et al., 2017) comprises seven positively worded items such as *I have been feeling useful*. Participants rated the frequency they have had those symptoms over the last 2 weeks (1 = *none of the time* to 5 = *all of the time*). The scores are summed to give a total score in the range of 7–35, with higher scores representing better mental health and wellbeing. Internal consistency of this measure in our study was good, with Cronbach’s α = 0.84.

### Procedure

After being informed about the nature and purpose of the study, students were given the option to provide electronic consent, and those who consented were then given access to the anonymous survey online. Most students completed the survey in their own time, while some completed the survey at the end of their classes. The surveys took approximately 15 minutes to complete, and students could earn partial credit towards a first-year course or to enter a prize draw (drawn each year) for one of ten $30 shopping vouchers. Following completion, participants were fully debriefed about the study. All methods and measures were approved by the University of Queensland human research ethics (low and negligible risk) committee (approval no. 2019001150).

### Statistical analysis

The two hypotheses will be tested in combination using a series of 2 × 4 between groups analyses of variance (ANOVAs), with two enrolment status subgroups (domestic and international students) and four cohorts (2019, 2020, 2021 and 2022). Where cohort effects are found, they will be followed up with post hoc tests to identify which cohorts are significantly different from which others. This Tukey’s test will account for six pairwise comparisons so to maintain a family-wise error rate of 0.05, an adjusted alpha of 0.011 will be used for determining the significance of post hoc tests.

## Results

### Data checking

Data from the four cohorts were downloaded and merged for analysis. A missing data analysis found fewer than 3% of data missing on any dependent variable; therefore, the analyses were conducted using deletion of cases with missing data. There was mild skewness on the depression (*Z*_skew_ = 9.06) and somatic distress (*Z*_skew_ = 6.78) variables, on which most of the respondents reported low scores while a few respondents scored in the elevated range of the scale. As ANOVA is robust to mild skewness in the data, no transformations were conducted on these variables.

### Main analyses

Descriptive statistics for the measured variables for each year cohort for domestic and international student groups are shown in Table 2. Two thirds (66.4%) of domestic students screened positive for mental health problems on PsyCheck in 2019, and this proportion increased to 75.5% in 2020, and returned to around two thirds again in 2021 and 2022. The proportion of international students screening positive for mental health problems on PsyCheck was lower than for domestic students in 2019 (46.3%) and this rose steeply to 66.6% in 2020, 64.6% in 2021 and 58% in 2022 (Table 2).

**Table 2.** Psychological distress and wellbeing in four cohorts (2019–2022) of the first-year domestic and international students at a metropolitan university in Australia.

	2019 cohort		2020 cohort		2021 cohort		2022 cohort	
	Domestic *N* = 289 Mean (SD)	International *N* = 162 Mean (SD)	Domestic *N* = 294 Mean (SD)	International *N* = 93 Mean (SD)	Domestic *N* = 266 Mean (SD)	International *N* = 99 Mean (SD)	Domestic *N* = 251 Mean (SD)	International *N* = 50 Mean (SD)
Positive screen for mental health	192 = 66.4%	75 = 46.3%	222 = 75.5%	62 = 66.6%	174 = 65.4%	64 = 64.6%	168 = 66.9%	29 = 58%
Depression (range: 0–4)	1.4 (1.4)	1.0 (1.3)	1.8 (1.5)	1.7 (1.5)	1.2 (1.4)	1.6 (1.5)	1.2 (1.4)	1.3 (1.3)
Anxiety (range: 0-4)	1.8 (1.4)	1.2 (1.2)	1.8 (1.3)	1.7 (1.2)	1.9 (1.3)	1.7 (1.3)	1.9 (1.3)	1.8 (1.1)
Somatic (range: 0–4)	1.6 (1.3)	1.1 (1.2)	1.6 (1.3)	1.5 (1.4)	1.6 (1.4)	1.2 (1.2)	1.6 (1.3)	1.2 (1.3)
Wellbeing (range: 7–35)	21.4 (4.0)	21.6 (4.1)	19.4 (3.1)	19.8 (4.0)	23.1 (4.6)	22.5 (4.9)	23.2 (4.8)	22.7 (4.6)

SD: standard deviation.

Key to measures: Positive screen for mental health was a score above 5 out of 20 on the PsyCheck screener. Depression was a sum of PsyCheck items 9, 11, 14, and 16. Anxiety was a sum of PsyCheck items 4, 5, 6 and 12; Somatic was a sum of PsyCheck items 1, 2, 7 and 19.

**Figure 1.**
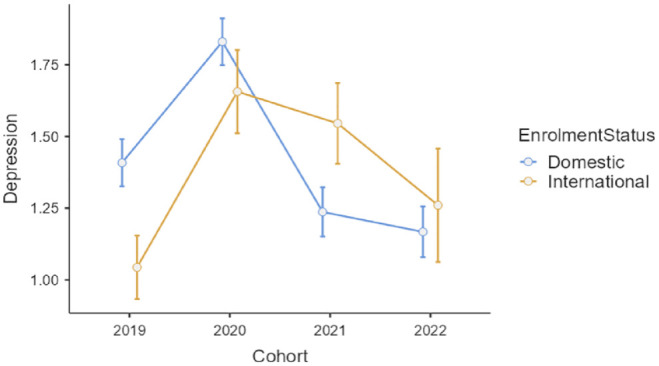
Depression scores for four cohorts of the first-year domestic and international students enrolled in an Australian metropolitan university (scale is 0 to 4 and bars are standard errors).

There was a statistically significant interaction on depression scores, *F*(3, 1494) = 3.60, *p* = 0.013, η_*p*_^2^ = 0.01 (see Figure 1). Post hoc tests confirmed that, after the depression peak in both enrolment groups in 2020, depression scores remained high for international students in 2021 (2020 to 2021 comparison *p* = 0.584), while depression scores returned to baseline levels in 2021 for domestic students (2020 to 2021 comparison *p* < 0.001).

**Figure 2.**
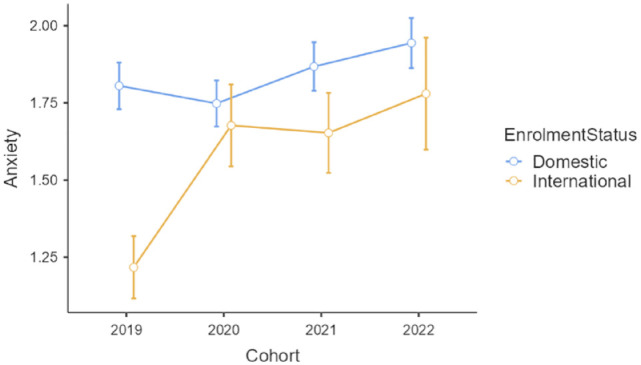
Anxiety scores for four cohorts of the first-year domestic and international students enrolled in an Australian metropolitan university (scale is 0 to 4 and bars are standard errors).

Anxiety scores showed a different pattern (see Figure 2), with a statistically significant interaction, *F*(3, 1492) = 2.75, *p* = 0.041, η_*p*_^2^ = 0.01. Post hoc testing indicated that although anxiety was worse overall for domestic students, the first wave of COVID-19 was associated with a greater increase in anxiety for international students (2019 to 2020 comparison *p* = 0.006) than for domestic students (2019 to 2020 comparison *p* = 0.59). Unlike the depression scores which were decreasing in both enrolment groups by 2022, the anxiety scores continued to increase in 2022.

**Figure 3.**
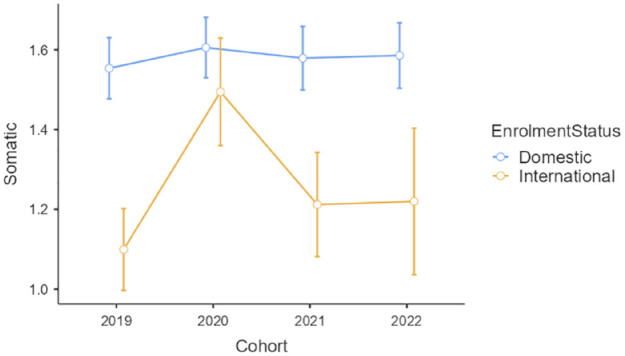
Somatic symptom scores for four cohorts of the first-year domestic and international students enrolled in an Australian metropolitan university (bars are standard errors).

A two-way ANOVA was performed to test the effects of enrolment status and cohort on somatic symptom scores, revealing there was no significant interaction, *F*(3, 1495) = 1.01, *p* = 0.385. There was a significant main effect of enrolment status on somatic symptoms, such that domestic students reported significantly more somatic symptoms of distress than their international counterparts, *F*(1, 1495) = 16.18, *p* < 0.001, η_*p*_^2^ = 0.01 (see Figure 3). The main effect of cohort was not significant, *F*(3, 1495) = 1.68, *p* = 0.170.

**Figure 4.**
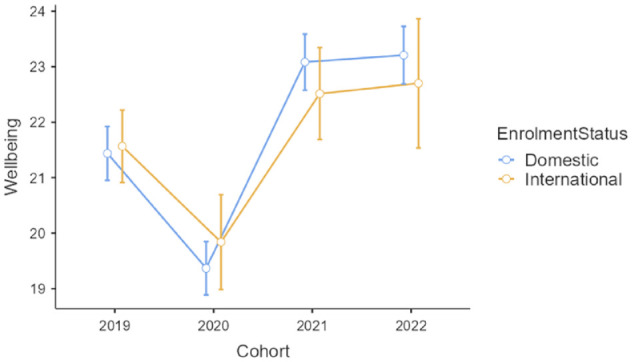
Warwick–Edinburgh Mental Well-being scores for four cohorts of the first-year domestic and international students enrolled in an Australian metropolitan university (bars are standard errors).

Finally, there was no significant interaction effect for wellbeing, *F*(3, 1491) = 0.95, *p* = 0.414, but there was a significant main effect of cohort, *F*(3, 1491) = 34.98, *p* < 0.001, η_*p*_^2^ = 0.07. Post hoc comparisons using the Tukey’s test confirmed the detrimental impact of the COVID-19 pandemic on students’ wellbeing, with scores in 2020 significantly lower than those in 2019 (*p* < 0.001), 2021 (*p* < 0.001) and 2022 (*p* < 0.001; see Figure 4). The effect of enrolment status on wellbeing was not significant, *F*(1, 1491) = 0.21, *p* = 0.649.

## Discussion

This study of four cohorts of first-year students enrolled at an Australian university provides clear evidence that the outbreak of the COVID-19 pandemic was associated with a spike in mental health problems in both domestic and international students. Even prior to COVID-19 (in 2019), the prevalence of domestic students screening positive for mental health problems was two thirds, consistent with rates of psychological distress reported in previous research (Rickwood et al., 2016; Stallman, 2010). This was a high rate of distress prior to the outbreak of the pandemic, indicating that previous mental health mitigation policies for university students may not have been sufficiently effective. Consistent with our first hypothesis, this rate increased to three quarters of the cohort in 2020. Among international students, the rate went from a half to two thirds of the cohort. We also observed a significant increase in depression and anxiety symptoms coinciding with the first wave of COVID-19. The first wave of COVID-19 was also associated with a significant decrease in wellbeing for all university students, which rebounded by 2022 to levels consistent with population scores reported in previous research (e.g. Ng Fat et al., 2017).

Interestingly, although somatic symptoms increased for international students during the first wave, there was no significant interaction of cohort and enrolment status observed on this variable. In fact, domestic students reported higher somatic symptoms overall than international students. This finding contrasts with previous research that found that Chinese people are more likely to somatise their experience of depression than Australian people (Chang et al., 2017). Nearly a quarter of our sample identified their ethnicity as North-East Asian (which includes Chinese), with smaller proportions identifying as South-East Asian (13.5%, including Singapore, Thailand, Malaysia and Indonesia) and Southern and Central Asian (6.1%).

Our second hypothesis – that the COVID-19 pandemic would impact on the mental health of international students more than domestic students – was not supported. In fact, across most variables and cohorts, domestic students reported worse mental health than their international student counterparts. Possible explanations for this include cultural issues around stigma related to disclosure of symptoms, as has been reported in a US study with 44,851 undergraduate students (Yeung et al., 2022). In this study, international students were less likely than domestic students to report a diagnosis of anxiety, comorbid depression and anxiety or other psychiatric diagnoses, yet international students were more likely to report suicide attempts and feeling overwhelmingly depressed (Yeung et al., 2022). Furthermore, Asian cultures tend to value emotional self-control and humility, which may result in depressive symptoms and an unwillingness to seek help from mental health professionals (Wong et al., 2014). With this international research in mind, it is difficult to know for sure whether international students in our study experienced lower levels of distress than their domestic peers or were simply less likely to report their experiences.

Indeed, in our study, depression scores were higher for international students than domestic students in 2021 and slower to return to pre-COVID levels. A possible reason for this finding is that during the pandemic, international borders were closed for much longer than other public health restrictions were in place, which impacted international students’ ability to arrive in Australia, and their ability to go home to see family and friends and return to study in Australia. This is also consistent with recent literature suggesting that the associated restrictions during the pandemic have halted the adaptation processes international students undergo in the transition to overseas study (e.g. Ploner, 2018), especially if they are from collectivist cultures, therefore increasing that sense of loneliness and social alienation. Indeed, a companion paper drawn from the same project as the current study found that levels of loneliness and sense of belonging at university were more problematic for domestic than international students in the 2019–2021 period. This study asked participants about 10 common causes of stress in each year cohort. The causes rated most highly were group assignments, studying for exams, maintaining a healthy lifestyle, friendship issues and mental health issues (Dingle et al., 2022).

In contrast with depression and wellbeing, which largely returned to pre-COVID levels by 2022, anxiety symptoms have continued to rise in 2021 and 2022. This is important because anxiety interferes with students’ capacity to learn and remember information (e.g. Eysenck et al., 2007). Possible causes of these increasing levels of anxiety among students include ongoing threats from high rates of COVID-19 infection, hospital admissions and deaths reported in the media (Mahat-Shamir et al., 2021), continued uncertainty about job security (Oswald et al., 2021), rising costs of living, shortages of affordable rental accommodation and environmental disasters (e.g. Gibbs et al., 2021; Hickman et al., 2021) that have occurred at an increasing rate over these same 4 years. It is also possible that we are seeing the effects of prolonged stress in the 2022 cohort – most of whom were in year 11 in 2020, so of all the cohorts they have since experienced the longest accumulative stress related to COVID-19 while also during their formative years of adolescence and young adulthood.

A strength of this study is the pre-COVID-19 time point, which allows a recent and highly relevant baseline of the mental health status of students entering the university against which we can compare the impact of the first and subsequent waves of COVID-19. However, there are also several limitations to note. We relied on convenience sampling methods, which may not have accurately represented the full first-year university population. Also, the Cronbach’s alpha values of the anxiety and somatic symptom subscales on the PsyCheck were only adequate, meaning that these values should be interpreted with caution. Nevertheless, there was a high degree of concordance across the measures used in the study, which increases our confidence in the effects found.

As noted in the National Framework for University Mental Health (Orygen, 2020), university settings play a key role in shaping and supporting students’ mental health and wellbeing. Students can take a proactive role in protecting their mental health by engaging in simple strategies such as socialising with others, eating well, sleeping well, participating in physical activity, implementing study skills and practising emotion regulation–particularly in group contexts with other students (Dingle, Hodges, et al., 2021; Ruiz-Hernández et al., 2022). Students believe that teachers play an influential role in their wellbeing through being available and supportive and by demonstrating competence and passion for their work (Eloff et al., 2021). Finally, the university administration can play a strategic role in implementing university-wide policies that support the development of student connections with their teachers and peers. With most Australian school-leavers going to university (Rickwood et al., 2016), these widespread and increasing levels of psychological distress are detrimental not just to individual students’ ability to achieve long-term success such as employment and financial security, but potentially to Australia’s future economic productivity (Productivity Commission, 2020).

To conclude, this study found clear evidence that the COVID-19 pandemic was associated with negative outcomes for mental health and wellbeing of university students. Domestic students reported worse mental health than international students across cohorts and measures, apart from depression which was higher in international students in 2021 and slower to return to baseline. These findings suggest that university students could benefit from a range of strategies and programmes to support their mental health during the pandemic and recovery to the ‘new normal’. The significant finding that both domestic and international students have higher anxiety levels than before the COVID-19 pandemic emphasises this and the need for effective prevention strategies to prevent further increases.
